# Objective evaluation of the acute effects of pelvic floor muscle training on vaginal dryness in postmenopausal women: a randomized controlled pilot study

**DOI:** 10.1007/s00404-025-08267-1

**Published:** 2026-01-12

**Authors:** Hatice Gulsah Kurne, Nebahat Uzunay, Doruk Cevdi Katlan, Turkan Akbayrak

**Affiliations:** 1 Division of Physiotherapy and Rehabilitation, Institute of Health Sciences, Hacettepe University, Ankara, Turkey; 2 Gynecology and Obstetrics, Istanbul Training and Research Hospital, Istanbul, Turkey; 3 Division of Fundamental Physiotherapy and Rehabilitation, Faculty of Physical Therapy and Rehabilitation, Hacettepe University, Ankara, Turkey

**Keywords:** Vaginal atrophy, Vaginal lubrication kit, Pelvic floor exercise

## Abstract

**Purpose:**

This study aimed to investigate the acute effects of pelvic floor muscle training (PFMT) on vaginal dryness in postmenopausal women using an objective measurement method within the framework of a randomized controlled trial.

**Methods:**

This randomized controlled pilot study included postmenopausal women who were allocated into two groups: a PFMT group (*n* = 45) and a control group (*n* = 58). The PFMT group performed a single set of exercises in the lithotomy position, consisting of 10 slow and 10 fast pelvic floor muscle contractions. The control group received no intervention.

The primary outcome was vaginal dryness, assessed objectively using the Vaginal Lubrication Kit (VLK). Secondary outcomes included Visual Analog Scale (VAS) scores for vaginal dryness, burning, and dyspareunia, as well as smear test results. Associations of baseline VLK and VAS scores with smear test findings were analyzed. Pre- and post-treatment differences, as well as between-group comparisons, were evaluated using parametric and non-parametric tests, with a 95% confidence interval.

**Results:**

Of the 105 women randomized, 103 completed the study. Baseline characteristics did not differ significantly between groups (*p* > 0.05). Following the intervention, the PFMT group demonstrated statistically significant improvements in VLK scores (*p* < 0.05). Baseline VAS scores were statistically significantly negatively correlated with the initial VLK score (VAS vaginal dryness, VLK r: −0.571, VAS vaginal burning, VLK r: −0.451, VAS dyspareunia, VLK r: −0.460, *p* < 0.05). In addition, the presence of atrophy in the pre-treatment smear test was found to be statistically significantly negatively correlated with the initial VLK score (r: −0.346, *p* < 0.05).

**Conclusions:**

PFMT significantly reduced vaginal dryness in postmenopausal women compared with the control group. It may be considered a potential treatment option for vaginal dryness. The VLK appears to be a promising objective tool for both assessing vaginal dryness and monitoring treatment outcomes in postmenopausal women. Although these findings are promising, they should be interpreted with caution given the pilot design and the short follow-up. Larger randomized trials are needed to confirm the long-term efficacy of PFMT and to validate the VLK as an objective outcome measure.

## What does this study add to the clinical work?

**Table:** 

This pilot randomized controlled study suggests that pelvic floor muscle training may acutely reduce vaginal dryness in postmenopausal women based on objective assessment. Additionally, the Vaginal Lubrication Kit may serve as a useful objective tool for evaluating vaginal dryness and short-term treatment effects in clinical settings.	

## Introduction

Vulvovaginal atrophy (VVA), resulting from estrogen deficiency during the postmenopausal period, is characterized by symptoms and findings associated with anatomical and physiological changes in the vulvovaginal tissues [1]. Involvement of the urogenital system leads to genitourinary syndrome of menopause (GSM), which encompasses a range of genital, sexual, and urinary symptoms [1]. Vaginal dryness is the primary symptom of GSM [2]. A study assessing its prevalence in postmenopausal women reported GSM in up to 90% of cases [3]. Furthermore, the decline in estrogen levels during menopause leads to the development of atrophy in estrogen receptor-sensitive tissues, contributing to vaginal laxity syndrome [4]. These estrogen deficiency-related syndromes are associated with the three primary categories of female sexual dysfunction as defined in the *Diagnostic and Statistical Manual of Mental Disorders* (DSM-5): interest/arousal disorder, orgasmic disorder, and genito-pelvic pain/penetration disorder [5, 6]. These chronic conditions, which tend to worsen with aging and the presence of comorbidities, negatively affect sexual satisfaction for both women and their partners [7]. Studies have reported the prevalence of sexual dysfunction in postmenopausal women to be approximately 17.7% in Northern Europe, 42.2% in Southeast Asia, and about 47.0% in Turkiye [8, 9].

Various hormonal and non-hormonal treatments are employed to manage GSM symptoms [10–17]. Since the 1960s, hormone replacement therapy (HRT), vaginal estrogen preparations, and vaginal moisturizers have been the most widely used treatments for GSM and remain first-line options in many settings [10–17]. By contrast, hyaluronic acid-based products, laser-based therapies, and pelvic floor muscle training (PFMT) have gained attention more recently—predominantly over the past decade—and their comparative effectiveness and long-term safety continue to be debated in the literature. Accordingly, both temporal trends and country-specific practice patterns influence treatment selection, with traditional hormonal and non-hormonal options remaining dominant, while newer approaches are still emerging. One of these treatment options is PFMT. However, the literature includes only a limited number of randomized controlled trials (RCTs) investigating the effects of PFMT on female sexual function. Although existing studies indicate that PFMT may improve sexual function, they present certain limitations [18, 19]. Furthermore, vaginal dryness has predominantly been assessed using subjective, self-reported scales [20, 21].

Limitations related to the objective measurement of vaginal dryness and study methodologies underscore the need for further research. To date, no study has investigated the acute effects of PFMT on vaginal dryness. Therefore, the aim of this randomized controlled trial was to examine the acute effects of PFMT on vaginal dryness in postmenopausal women. This study is the first to objectively assess vaginal dryness using a newly developed method, the Vaginal Lubrication Kit (VLK). The primary objective was to evaluate the acute effect of PFMT on vaginal dryness, while the secondary objective was to assess the correlation of VLK scores with Visual Analog Scale (VAS) ratings for dryness, burning, and dyspareunia, as well as with smear test results.

## Methods

### Study design

This study was conducted as a randomized controlled pilot trial with two parallel groups to evaluate the acute effect of PFMT on vaginal dryness in postmenopausal women. The study protocol was approved by the Clinical Research Ethics Committee of the hospital (Date: December 22, 2023; approval number: 360) and registered at ClinicalTrials.gov (NCT06294197). The study was carried out between January 2024 and August 2024 at the Istanbul Training and Research Hospital. The protocol adhered to the principles of the Declaration of Helsinki and received local ethics committee approval. Informed consent was obtained from all participants prior to enrollment.

### Participants

Postmenopausal, sexually active women were included in the study. Inclusion criteria were willingness to participate, being amenorheic for at least 12 months, and being sexually active. Exclusion criteria included active vaginal or urinary infection, history of active malignancy, current radiotherapy or chemotherapy, use of hormone replacement therapy within the past year, use of local estrogen, coitus within the past 2 days, stage 2 or higher pelvic organ prolapse, history of mesh surgery, and the presence of diseases or medications known to cause vaginal dryness (e.g., Sjögren’s syndrome, lichen planus, lichen sclerosus, use of antidepressants or antihistamines). Participants were recruited at Istanbul Training and Research Hospital. Patients were chosen among ones who were planned to undergo routine smear testing.

### Randomization and blinding

Participants were randomly assigned to two groups—the PFMT group (*n* = 47) and the control group (*n* = 58)—using simple randomization conducted by a researcher. This was a single-blind study; the assessor and the outcome evaluator (Gynecologist, MD, Nebahat UZUNAY) were blinded to group allocation. The physiotherapist delivering the intervention was not blinded due to the nature of the study.

### Intervention

The intervention group received PFMT administered by a physiotherapist specialized in women's health. The training focused on enabling participants to isolate their pelvic floor muscles (PFM) and perform effective contractions and relaxations. The intervention consisted of one set of slow and fast contractions performed in the lithotomy position with vaginal palpation. Specifically, one set included 10 repetitions of slow pelvic floor muscle contractions followed by 10 repetitions of fast contractions. Each session lasted approximately 10 min. The control group received no intervention.

### Descriptive and outcome measures

At baseline, demographic, physical, and obstetric characteristics were recorded (Table 1).

**Table 1:** Characteristics of the cases

	PFMT group (*n* = 45)	Control group (*n* = 58)	*p*
Age (year), (IQR)	56.6 (46–70)	56 (37–74)	0.968
BMI (kg/m^2^), (IQR)	29.6 (21–44)	27.5 (16–39)	0.035
Employment status, % (*n*)	100% (45)	100% (58)	0.958
Employed	31.1 (14)	19 (11)	0.958
Unemployed	44.4 (20)	70.7 (41)	0.958
Retired	24.4 (11)	10.3 (6)	0.958
Education, % (*n*)	100% (45)	100% (58)	0.117
Illiterate	0	3.4 (2)	0.117
Literate	4.4 (2)	5.2 (3)	0.117
Primary school graduate	46.7 (21)	63.8 (37)	0.117
High school graduate	31.1 (14)	19 (11)	0.117
University graduate	13.3 (6)	5.2 (3)	0.117
Postgraduate graduate	4.4 (2)	3.4 (2)	0.117
Marital status, % (*n*)	100% (45)	100% (58)	0.582
Single	0	0	0.582
Married	82.2 (37)	86.2 (50)	0.582
Has a partner	17.8 (8)	13.8 (8)	0.582
Parity, (IQR)	2.6 (0–7)	2.9 (0–8)	0.652
Number of vaginal births, (IQR)	1.7 (0–6)	2.4 (0–8)	0.036
Number of cesarean births, (IQR)	0.5 (0–2)	0.2 (0–3)	0.016
Type of menopause onset, % (*n*)	100% (45)	100% (58)	0.302
Natural menopause	93.3 (42)	94.8 (55)	0.302
Surgical menopause	6.7 (3)	5.1 (3)	0.302
Age at menopause onset	47.9 (38–55)	47.3 (36–60)	0.556
Duration of menopause	8.2 (1–23)	8.7 (1–34)	0.689
Smear atrophy, % (*n*)	50.48	49.52	0.912
History of gynecological surgery, % (*n*)	8.9 (4)	6.9 (4)	0.709
History of hormone therapy % (*n*)	4.4 (2)	0	0.107
Frequency of sexual intercourse (per month), (IQR)	4 (1–8)	4 (1–16)	0.674
VAS dryness (cm), (IQR)	4.8 (0–10)	4.1 (0–10)	0.376
VAS burning (cm), (IQR)	3.7 (0–10)	3.7 (0–10)	0.733
VAS dyspareunia(cm), (IQR)	3.4 (0–10)	3.1 (0–10)	0.818

*BMI* body mass index, *IQR* interquartile range, *PFMT* pelvic floor muscle training, *VAS* visual analog scale

The primary outcome was the VLK result. The VLK, developed to objectively assess vaginal dryness, was adapted from Schirmer’s test for eye dryness [22]. In this study, Schirmer’s test strips were modified for vaginal application. Strips measuring 5 mm × 40 mm and lacking blue dye were folded at the 5 mm mark within the sterile package to prevent the passage of fluids other than vaginal lubrication. The strip was placed 2 cm proximal to the Carunculae Hymenalis using sterile forceps or by holding the tip and remained in place for 5 min. The distance of wetness was measured immediately upon removal. A patent application for the test strip was filed with the Turkish Patent Office (Application No. 2025/007314). In both groups, the VLK assessment was performed at baseline. In the intervention group, pelvic floor muscle exercises lasted approximately 10 min, and the VLK measurement was repeated immediately thereafter. In the control group, the VLK measurement was conducted twice: at baseline and again 5 min after the initial assessment.

Secondary outcomes included correlations between VLK scores and Visual Analog Scale (VAS) values for vaginal dryness, burning, and dyspareunia, as well as smear test results. The VAS was used to quantify GSM symptoms along a 10-cm horizontal line, where 0 indicated no symptom and 10 indicated an unbearable symptom; scores were determined by measuring the distance from zero to the participant’s mark. Smear test findings focused on estrogen-related changes in the cervicovaginal stratified squamous epithelium [23].

### Data analysis

Data analysis was conducted using SPSS version 23 (IBM SPSS, Chicago, IL, USA). Parametric and non-parametric distributions were assessed. Quantitative data are reported as mean and standard deviation (SD). The paired sample *T* test was applied to dependent parametric data, while the Wilcoxon test was used for non-parametric dependent data. Between-group comparisons of non-parametric data were performed using the Mann–Whitney *U* test. Correlations between baseline VLK scores and secondary outcome measures (VAS scores and presence of atrophy in smear test) were analyzed using Spearman’s correlation coefficient. Effect sizes were calculated using G*Power (version 3.1.9.7) and categorized as > 0.80 (large), 0.5–0.8 (medium), or < 0.5 (small) [23]. For a one-sided hypothesis test, the required sample size was 82 (41 per group) to achieve 85% power at a 5% type I error rate. To account for a 10% dropout rate, 98 participants were enrolled. Statistical Significance was set at *p* < 0.05.

## Results

**Fig. 1:**
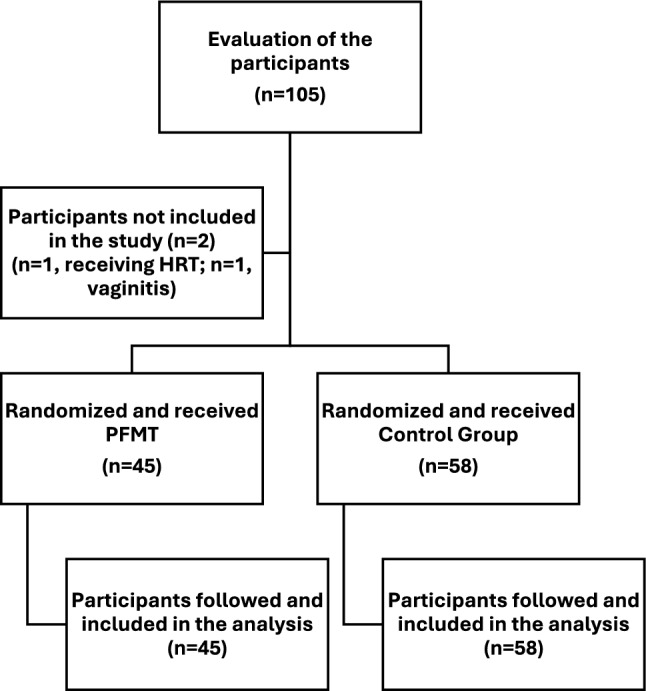
Flow chart

A total of 105 postmenopausal women were assessed for eligibility, and 103 were included in the study. Two individuals were excluded for not meeting the inclusion criteria due to vaginitis and ongoing hormone replacement therapy. Forty-five women were randomized to the PFMT group and 58 to the control group (Fig. 1).

The baseline characteristics of the participants were not clinically different between groups (Table 1). The mean age of the participants was 56 years (PFMT group IQR: 46–70; control group IQR: 37–74). The mean age at menopause onset was 47 years in both groups (PFMT group IQR: 38–55; control group IQR: 36–60), and the mean duration of menopause was 8 years (PFMT group IQR: 1–23; control group IQR: 1–34).

The primary outcome, the VLK score, was similar between the intervention and control groups at baseline (IG = 9.8 mm, CG = 8.6 mm, *p* > 0.05). Post-treatment comparisons revealed a statistically significant difference between the groups (mean difference = 3.49 mm; 95% CI; *p* < 0.000) (Table 2). Following treatment, vaginal lubrication scores in the intervention group increased, indicating an 87% improvement.

**Table 2:** Primary outcomes according to randomized treatment adjusted for baseline

	PFMT group (*n*:45)			Control group (*n*:58)			D1–D2	*p* value
	BT	AT	D1	BT	AT	D2		
VLK score (mm)	9,82 ± 5,48	13,48 ± 5,90	3,73 ± 3,40	8,62 ± 5,26	8,79 ± 5,10	0,24 ± 1,94	3,49 ± 1,50	
VAS score, vaginal dryness (cm)	4,69 ± 3,55	2,47 ± 2,18	−2,22 ± 1,86	4,26 ± 3,13	4,18 ± 3,20	−0,09 ± 0,66	−2,13 ± 1,20	0,000

*PFMT* pelvic floor muscle training, *VLK* vaginal lubrication kit, *VAS* visual analog scale, Values inside the boxes (X ± SD) mean with standard deviations, *BT* before treatment, *AT* after treatment, D1 difference of PFMT group (AT–BT), D2 difference of control group (AT–BT)

VAS scores for the secondary outcome measures of vaginal dryness (VD), vaginal burning (VB), and dyspareunia (D) were calculated. All VAS scores demonstrated statistically significant negative correlations with baseline VLK scores, which were of moderate strength (VD, VLK r = −0.571; VB, VLK r = −0.451; D, VLK r = −0.460; *p* < 0.000). The presence of atrophy in the smear test, another secondary outcome measure, was calculated as a percentage, with atrophic changes detected in 50.48% of participants. A statistically significant negative correlation was also observed between the presence of atrophy in the smear test and baseline VLK scores, although this relationship was of weak strength (r = −0.346, *p* < 0.05).

Baseline VLK scores were found to be significantly lower in participants with the presence of atrophy on the smear test when compared with those without atrophy (Atrophy group mean VLK: 7.53 mm, No atrophy group mean VLK: 10.80 mm). This finding indicates that VLK scores have a strong association with smear test results, reinforcing the objectivity of measurement (Table 3).

**Table 3:** Relationship between baseline VLK scores and presence of atrophy in the smear test

	Mean of initial VLK score (mm)	*p* value
Participants with the presence of atrophy	7.53	0.000
Participants without the presence of atrophy	10.80	0.000

*VLK* vaginal lubrication kit, *mm* millimeter

## Discussion

This study demonstrates the acute effects of PFMT, a treatment approach for vaginal dryness—a key symptom of GSM in postmenopausal women—emphasizing the significance of muscle training. To date, this is the first study to investigate the acute effects of PFMT on vaginal dryness in this population. Vaginal dryness was objectively assessed using the VLK, a novel measurement tool developed specifically for this purpose. Furthermore, within the framework of a randomized controlled trial, PFMT was shown to produce positive acute effects on vaginal dryness in postmenopausal women.

The participants, who were comparable in terms of demographic, physical, and medical characteristics potentially associated with vaginal dryness, enabled within-group and between-group comparisons to be interpreted independently of these confounding factors. The primary outcome measure, the VLK score, demonstrated significant within-group improvement in the PFMT group and significant between-group differences with a large effect size, indicating a clinically meaningful reduction in vaginal dryness. Notably, 87% of participants in the intervention group experienced a decrease in vaginal dryness in acute post-treatment assessments.

Secondary outcome measures—VAS scores for vaginal dryness, burning, and dyspareunia—showed significant negative correlations with baseline VLK scores, indicating that higher symptom severity was associated with lower levels of vaginal lubrication as measured by the VLK. In addition, the presence of atrophy on smear tests demonstrated a significant negative correlation with baseline VLK scores, suggesting that atrophy increased in parallel with increased vaginal dryness. In this study, atrophic changes were detected in 50.48% of participants.

Multiple treatment options are available to manage GSM symptoms in postmenopausal women, including hormonal therapies, fractional CO₂ laser applications, hyaluronic acid treatments, radiofrequency therapies, lubricants/moisturizers, and PFMT [10–17, 24, 25]. However, limitations such as contraindications to hormonal therapy, delayed onset of effects with laser treatments, and the short-term relief provided by lubricants can hinder effective symptom management. PFMT offers several advantages, including low cost, absence of side effects, immediate symptom relief, long-term benefits, and suitability for use outside clinical settings.

Previous studies on GSM treatment have reported intervention durations ranging from 1 to 3 months [10–17, 24, 25]. In a randomized trial evaluating CO^2^ laser therapy, no acute outcomes were reported; however, significant improvements in VAS scores were observed at 30 days (*p* < 0.0001) [26]. Similarly, studies examining PFMT or ospemifene have demonstrated improvements only after several months [13, 27]. The present study is the first to evaluate the acute outcomes of PFMT using an objective measurement method, showing that PFMT can acutely reduce vaginal dryness. This finding is particularly significant in terms of treatment efficacy, time efficiency, and cost-effectiveness.

PFMT aims to strengthen the pelvic floor muscles, enhance vascularization, and regulate muscle tone and function [12, 27]. These effects support tissue elasticity and the vaginal epithelium, and may contribute to improved sexual satisfaction [27]. A single-arm study demonstrated that PFMT increased blood flow in the internal pudendal and dorsal clitoral arteries, as measured by Doppler ultrasound [27]. In addition, PFMT may influence clitoral position and sensitivity, potentially enhancing arousal and orgasm [28]. Moreover, PFMT may reduce the need for higher cost diagnostic and therapeutic procedures [29].

Vaginal dryness is a significant health concern in GSM and contributes to sexual dysfunction in both women and their partners [3, 7]. Despite its high prevalence, many postmenopausal women do not report vaginal or sexual health concerns, often due to cultural or social factors [29–32]. Consistently, in the present study, the prevalence of sexual dysfunction was found to be 69.9%, supporting previous evidence that postmenopausal women with GSM symptoms are approximately four times more likely to experience sexual dysfunction compared to those without GSM symptoms [33].

Few randomized trials have evaluated the effects of PFMT on sexual function [18, 19]. These studies primarily relied on subjective scale scores, and their methodological limitations underscore the need for objective assessment tools. The present findings are consistent with previous research demonstrating the positive long-term effects of PFMT on sexual function [34, 35]. Unlike existing indices, such as the Vaginal Health Index, the VLK offers numerical, objective data specific to vaginal dryness [36–38]. Nevertheless, the optimal frequency, intensity, and duration of PFMT for sustained improvements in vaginal dryness remain undefined. Prior 12-week structured PFMT protocols have reported promising outcomes; Mercier et al. demonstrated clinically meaningful improvements in vaginal dryness, dyspareunia, and sexual quality of life using a program of one supervised 60-min session weekly combined with 5 days of home exercises [27].

The strengths of this study include the novel use of the VLK as an objective assessment tool, the implementation of an individualized PFMT program supervised by an experienced physiotherapist, and the use of independent outcome assessment to minimize bias. Limitations include challenges in achieving full blinding due to the nature of the intervention, and the lack of comprehensive psychometric evaluation (e.g., reliability and validity testing) of the VLK, which was introduced here for the first time. As a pilot trial, these findings provide a foundation for future randomized studies with long-term follow-up and further validation of the VLK. Although the observed differences were statistically significant, their clinical relevance remains uncertain. Future adequately powered randomized controlled trials are required to establish whether these changes represent clinically meaningful improvements and to determine minimal clinically important difference (MCID) thresholds for the VLK measurements.

## Conclusion

In this randomized controlled pilot study, a single session of supervised pelvic floor muscle training (PFMT) significantly reduced self-reported vaginal dryness in postmenopausal women compared with controls. These findings indicate that PFMT may represent a promising non-pharmacological strategy in the management of genitourinary syndrome of menopause (GSM), specifically targeting vaginal dryness. Furthermore, the Vaginal Lubrication Kit (VLK), adapted from the Schirmer test, demonstrated potential as an objective tool for assessing vaginal lubrication and monitoring treatment responses.

However, given the pilot design, small sample size, and short follow-up period, the results should be interpreted with caution. Further large-scale, adequately powered randomized controlled trials are warranted to (i) establish the reliability and validity of the VLK, (ii) determine clinically meaningful thresholds for change, and (iii) evaluate the optimal frequency, intensity, and duration of PFMT to achieve sustained improvements. Until such evidence becomes available, PFMT can be considered a complementary or alternative approach, with the VLK offering a novel means of objective outcome evaluation.

## Data Availability

Data are provided within the manuscript or the supplementary information files.
